# A case of phacolytic glaucoma with anterior lens capsule disruption identified by scanning electron microscopy

**DOI:** 10.1186/1471-2415-14-133

**Published:** 2014-11-19

**Authors:** Woong-Sun Yoo, Byeong-Jae Kim, In-Young Chung, Seong-Wook Seo, Ji-Myong Yoo, Seong-Jae Kim

**Affiliations:** Department of Ophthalmology, Gyeongsang National University, Colleage of Medicine, Jinju, South Korea; Gyeongsang Institute of Health Science, Gyeongsang National University, Jinju, South Korea; Department of Ophthalmology, School of Medicine, Institute of Health Science, Gyeongsang National University Hospital, 79 Gangnam-ro, Jinju, Gyeongnam 660-702 South Korea

**Keywords:** Phacolytic glaucoma, Lens capsule, Scanning electron microscopy (SEM)

## Abstract

**Background:**

Phacolytic glaucoma is induced by lens protein or macrophages that have leaked through a macroscopically intact anterior lens capsule. Here, we report a case of phacolytic glaucoma with anterior lens capsule disruptions visualized by scanning electron microscopy (SEM).

**Case presentation:**

A 71-year-old man was referred to our institute for increased intraocular pressure (IOP) in the right eye. Slit-lamp biomicroscopic examination revealed corneal edema, the presence of inflammatory cells and iridescent crystalline in the anterior chamber, and a hypermature cataract in the right eye. Despite treatment with topical glaucoma medication (0.15% brimonidine, 1% brinzolamide/0.5% timolol, and 0.03% bimatoprost) and systemic mannitol, his IOP remained uncontrolled. Light microscopy was used to examine the aqueous humor obtained via anterior chamber paracentesis and the anterior lens capsule obtained via intracapsular cataract extraction (ICCE), which revealed that the anterior lens capsule was intact. However, SEM revealed full-thickness disruptions in the anterior lens.

**Conclusion:**

This is the first reported case of phacolytic glaucoma with disruptions of the anterior lens capsule confirmed by SEM.

## Background

Phacolytic glaucoma is open-angle glaucoma induced by mature or hypermature cataract. During this condition, the soluble contents of the lens leak into the anterior chamber and obstruct trabecular outflow. The lens capsule in phacolytic glaucoma appears grossly intact or occasionally shows spontaneous non-traumatic defects [1–3]. Here, we present a case of phacolytic glaucoma in which anterior lens capsule disruptions were identified by SEM and that was successfully treated.

## Case presentation

**Figure 1:**
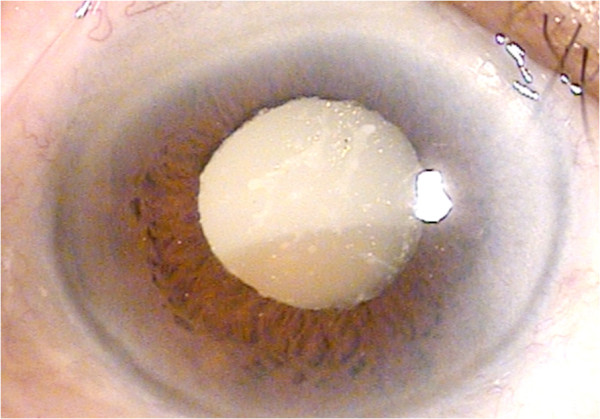
**Photograph of slit-lamp examination at initial visit.** Slit-lamp examination revealed corneal edema, iridescent crystalline in the anterior chamber, and hypermature cataract in the right eye.

The patient was a 71-year-old man with no systemic or ophthalmologic disorders. He developed ocular pain and decreased visual acuity of the right eye abruptly over 2 weeks before visiting the local clinic. He was referred to a tertiary referral center for uncontrolled IOP. His visual acuity was hand movement in the right eye and 1.0 in the left eye. IOP was 50 mmHg and 12 mmHg in the right and left eyes, respectively. Slit-lamp examination revealed corneal edema, the presence of inflammatory cells and multiple iridescent crystalline in the anterior chamber, and hypermature cataract in the right eye (Figure 1), while the left eye showed a mild nuclear cataract. Gonioscopic examination revealed open angles in both eyes and the presence of iridescent crystalline in the trabecular meshwork of the right eye. He was treated with instillation of topical glaucoma medication (0.15% brimonidine, 1% brinzolamide/0.5% timolol, and 0.03% bimatoprost) and systemic mannitol. IOP remained high despite intensive anti-glaucoma therapy. We made a diagnosis of phacolytic glaucoma and planned to perform extracapsular cataract extraction (ECCE). However, we found zonulysis in nearly two third of the lens. Because of this, we choose to perform ICCE rather than ECCE. After performing anterior chamber paracentesis (0.3 mL) for diagnostic purposes, ICCE was performed using lens capsular forcep and spoon under retrobulbar anesthesia through a 10-mm superior corneoscleral incision.

The acquired lens and capsule were fixed in 2% glutaraldehyde, embedded in paraffin, and then sectioned at a thickness of 5 μm. Aqueous humor and serial sections of the lens capsule were stained with hematoxylin and eosin and examined under an Olympus BX51 light microscope (Olympus Corporation, Tokyo, Japan). Ultrathin sections (50 μm) were stained with uranyl acetate and lead citrate, and then examined using a Zeiss Libra 120 electron microscope (Carl Zeiss SMT AG Company, Oberkochen, Germany).

**Figure 2:**
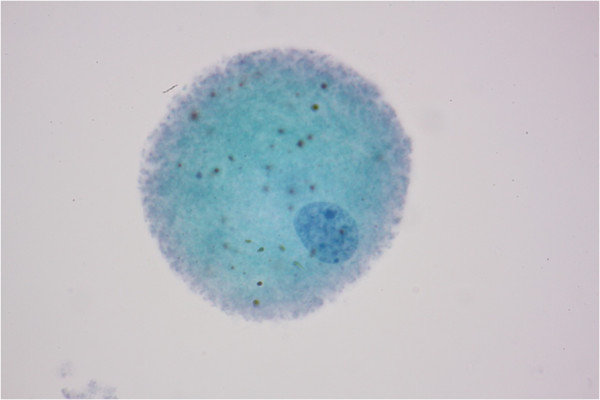
**Histological examination of aqueous humor by light microscopy.** Light microscopic examination of the aqueous humor revealed the presence of macrophages with multiple pigmented cytoplasmic material (hematoxylin-eosin staining, magnification 1000×).

**Figure 3:**
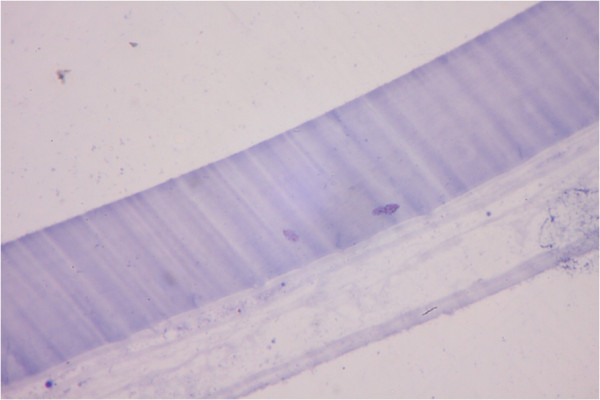
**Histological examination of the anterior lens capsule by light microscopy.** Light microscopic examination of the anterior lens capsule revealed intact histological appearance with no disruptions (hematoxylin-eosin staining, magnification 400×).

**Figure 4:**
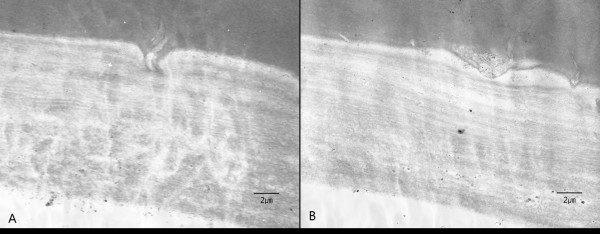
**Scanning electron microscopy of the anterior lens capsule.** Histopathologic findings showed many full-thickness dehiscences and grooves in the central portion of the anterior lens capsule **(A)**, while the peripheral portion had an intact appearance **(B)** (magnification 3500×).

In the aqueous humor, macrophages with multiple pigmented cytoplasmic materials believed to be lens protein were noted (Figure 2). Stained sections of the anterior lens capsule revealed intact structures by light microscopy (Figure 3). However, by scanning electron microscopic examination, the center of the anterior lens capsule showed full-thickness loss of tissue integrity with multiple grooves (Figure 4A) unlike the intact peripheral portion of the anterior lens capsule (Figure 4B).

Three days after ICCE, corneal edema had decreased with a moderate cellular inflammatory reaction in the anterior chamber. The patient was discharged and treated with topical steroid. At postoperative 2 months, corneal edema had disappeared and no cellular inflammatory reaction was noted in the anterior chamber. In the right eye, IOP was 15 mmHg without anti-glaucoma therapy, and best-corrected visual acuity increased to 0.4.

## Conclusions

Cataract changes in the lens can lead to glaucoma induced by obstruction of the trabecular meshwork with lens protein and macrophages, lens particles, or inflammatory cells stemming from an immune response. Phacolytic glaucoma is open-angle glaucoma induced by leakage of soluble contents into the anterior chamber by a hypermature or mature cataract. Unlike lens particle glaucoma, which often has lens fragments in the aqueous humor after capsular disruption, phacolytic glaucoma occurs with a grossly intact capsule and absence of lens particles [3]. However, the pathogenesis of phacolytic glaucoma is not fully understood. The mechanisms underlying the association between the presence of soluble contents and increased IOP remain under debate. One theory suggests that after leakage of its soluble contents, the aqueous humor becomes saturated with calcium oxalate and cholesterol crystals, which are found as hyperrefringent particles in the anterior chamber. At the same time, the obstruction of the trabecular meshwork with heavy molecular weight proteins from the lens and phagocytic macrophages leads to a characteristically severe elevation in IOP [4]. Alternatively, Mavrakanas et al [5] suggests two forms of phacolytic glaucoma: acute onset and gradual onset. Acute onset phacolytic glaucoma is caused by rapid leakage of liquefied lens protein into the aqueous humor through tiny spontaneous ruptures of the anterior lens capsule, without the presence of macrophages. Gradual onset phacolytic glaucoma is characterized by the presence of macrophages in the aqueous humor induced by an immunologic reaction to lens protein through an intact lens capsule [6]. However, whether the lens capsule is indeed intact in patients with phacolytic glaucoma has not yet been confirmed by electron microscopy.

Recently, studies reported the characterization of the lens capsule by electron microscopy. In one study, the anterior lens capsule was described in patients with Alport syndrome based on electron microscopic analysis, and data showed no macroscopic anterior capsule rupture or tear in any of the patients by slit-lamp examination. Although light microscopy was not used in that study, electron microscopic examination of the anterior lens capsule revealed that the inner two-thirds of the anterior capsule had several vertical dehiscences [7]. Therefore, we hypothesized that the lens capsule of the current patient with phacolytic glaucoma may have ultrastructural disruption without macroscopically visible defects.

In our case, clinical diagnosis was phacolytic glaucoma which is different from lens particle glaucoma that have macroscopic abruption in anterior capsule of the lens. However, SEM revealed full-thickness loose capsular tissue and multiple grooves, suggesting that lens protein had leaked through the disruptions and caused an immunologic reaction or direct action on the trabecular meshwork. These findings suggest that phacolytic glaucoma and certain cases of lens particle glaucoma that occur with spontaneous capsule rupture may have similar disease mechanisms.

There are some limitations in our report. First, electron microscopy has intrinsic limitations, such as the potential presence of artifacts from sample preparation. Second, lens capsule might be traumatized by instrument during the surgery. However, we performed ICCE rather than ECCE, damage in capsule of the lens would be minimized. Finally, our study has the limitation of being a single case report. In light of our results, we plan to increase the sample size to confirm our findings in additional patients with phacolytic glaucoma.

Despite the limitations of the study, to the best of our knowledge, this is the first report of SEM finding of anterior lens capsule disruption in a phacolytic glaucoma patient, and this finding may be helpful to better understand the mechanism underlying phacolytic glaucoma and lens particle glaucoma.

## Consent

Written informed consent was obtained from the patient for publication of this case report and any accompanying images. A copy of the written consent is available for review by the Editor of this journal.

## Abbreviations

ECCE: Extracapsular cataract extraction
ICCE: Intracapsular cataract extraction
IOP: Intraocular pressure
SEM: Scanning electron microscopy
